# SOCIAL PARTICIPATION AND DEPRESSION AMONG HONG KONG OLDER ADULTS: THE MODERATING ROLE OF DIGITAL ENGAGEMENT

**DOI:** 10.1093/geroni/igad104.1383

**Published:** 2023-12-21

**Authors:** Chang Liu, Xue Bai, Chi-Ko Lee

**Affiliations:** The Hong Kong Polytechnic University, Hong Kong, Hong Kong; The Hong Kong Polytechnic University, Hong Kong, Hong Kong; The Hong Kong Polytechnic University, Hong Kong, Hong Kong

## Abstract

Depression is a significant public health issue among older adults. Active social participation is crucial for older people to promote mental well-being. Previous studies mainly focused on the impacts of offline social activities on older people’s mental health, despite that digital participation has substantially changed their patterns of social participation in the context of Covid-19 pandemic. Extending the use of Activity Theory to digital context, this study investigated how social participation in three types of activities (i.e., formal, informal and solitary activities) and digital engagement independently and jointly influenced mental health among Hong Kong older adults. Data from 5,007 participants were drawn from the baseline survey of the Panel Study of Active Ageing and Society, a biannual longitudinal survey conducted with a representative sample of ageing Chinese adults aged 50 years and above in Hong Kong. Exploratory factor analysis, ordinary least squares regression analysis and mediation analysis were conducted. The results showed that participation in informal and solitary activities was related to less depressive symptoms, while participation in formal activities was related to more depressive symptoms but higher life satisfaction. Although the main effect of digital engagement on depressive symptoms was not significant, more digital engagement reduced the negative effect of formal activities and decreased the effect of informal activities in reducing depressive symptoms. The findings have implications for policy-making and intervention development to encourage and balance social and digital participation among ageing populations.

